# Influence of satellite-derived rainfall patterns on plague occurrence in northeast Tanzania

**DOI:** 10.1186/1476-072X-9-60

**Published:** 2010-12-13

**Authors:** Annekatrien Debien, Simon Neerinckx, Didas Kimaro, Hubert Gulinck

**Affiliations:** 1 Department of Earth and Environmental Sciences, Katholieke Universiteit Leuven, Celestijnenlaan 200E, Leuven, Belgium; 2 Evolutionary Ecology Group, Department of Biology, Universiteit Antwerpen, Groenenborgerlaan 171, B-2020 Antwerp, Belgium; 3 Department of Agricultural Engineering and Land Planning, Sokoine University of Agriculture, P.O. Box 3003, Morogoro, Tanzania

## Abstract

**Background:**

In the tropics, rainfall data are seldom accurately recorded, and are often discontinuous in time. In the scope of plague-research in northeast Tanzania, we adapted previous research to reconstruct rainfall patterns at a suitable resolution (1 km), based on time series of NDVI: more accurate satellite imagery was used, in the form of MODIS NDVI, and rainfall data were collected from the TRMM sensors instead of in situ data. First, we established a significant relationship between monthly rainfall and monthly composited MODIS NDVI. The established linear relationship was then used to reconstruct historic precipitation patterns over a mountainous area in northeastern Tanzania.

**Results:**

We validated the resulting precipitation estimates with in situ rainfall time series of three meteorological stations located in the study area. Taking the region's topography into account, a correlation coefficient of 0.66 was obtained for two of the three meteorological stations. Our results suggest that the adapted strategy can be applied fruitfully to estimate rainfall variability and seasonality, despite the underestimation of overall rainfall rates. Based on this model, rainfall in previous years (1986) is modelled to obtain a dataset with which we can compare plague occurrence in the area. A positive correlation of 82% is obtained between high rainfall rates and plague incidence with a two month lag between rainfall and plague cases.

**Conclusions:**

We conclude that the obtained results are satisfactory in support of the human plague research in which this study is embedded, and that this approach can be applied in other studies with similar goals.

## Background

Climate and vegetation are strongly linked. In fact, climate would determine global land cover if anthropogenic interferences did not exist [1]. NDVI, the Normalised Difference Vegetation Index, is the most commonly used index of greenness derived from multispectral remote sensing data, and is used in several studies on vegetation, since it has been proven to be positively correlated with density of green matter [2-6] in [7]. Several studies examining the strength of the vegetation-precipitation relationship yield converging results, and state that NDVI is positively correlated with precipitation [8-11]. However, the magnitude of this correlation and the eventual time lag present between those two variables differ between studies. Despite sometimes large differences between established relationships depending on location, they all agree on the importance of the incorporation of a time lag in this relationship. This time lag can be explained by a delay in response of vegetation growth to changing soil moisture levels after rainfall events [11,12].

The strength of the relationship between NDVI and rainfall is influenced by many factors, the most important being land cover and the prevailing climate in the study area [9,10,13]. Different land cover types generate differences in correlation due to the fact that the same NDVI may represent different vegetation states in other vegetation communities. When taking land cover type into account in the modeling of the vegetation response to precipitation, it is generally found that correlations are weaker in forests than in grasslands [9-11]. Li [10], for example, finds the correlation coefficients of this relationship increasing over a typological sequence of land cover types: evergreen forest, deciduous forest, scrubs and desert vegetation, and steppe and savannah. The most obvious explanation is the difference in rooting system between the two vegetation types: trees have access to deeper soil layers, in which soil moisture does not decline as fast as in the upper soil layers [9] than shrubs, herbs, and grasses, which only reach more superficial soil layers.

The importance of the overall climate on the relationship between precipitation and NDVI has been observed in a number of studies [13-16]. Indeed, the correlation between NDVI and precipitation is stronger in regions where rainfall is relatively scarce (up to 650 mm) [17], since water availability only determines the vegetation vigour until a certain threshold, after which the extra water will not be used as efficiently. This is also illustrated by Camberlin *et al*. [18], who have determined the NDVI-precipitation relationship in Africa, and find the strongest correlations in semi-arid areas, with an annual rainfall between 200 and 600 mm. Since our study area lies in and around the Usambara and Pare Mountains in northeastern Tanzania, climate will be heavily influenced by the topography of the area. The effect of elevation on precipitation can be partially explained by the forced convection that occurs in mountainous areas [19]. The mountains in northeast Tanzania rise high above the surrounding Maasai plains, so clouds are forced to rise, causing them to cool, and shed rain at the side of the mountain facing the predominant winds. This leads to a complex pattern of wet and dry slopes in the northeast Tanzanian mountains.

The lack of a network of accurately recorded rainfall time series in the tropics is a problem faced by studies on multiple issues [17]. The Tropical Measuring Rainfall Mission is designed to measure rainfall rates in the tropics [20]. However, despite the fact that TRMM provides accurate spatial and temporal measurements of rainfall over the tropics, it only gives information at coarse resolutions (0.25° × 0.25° resolution) [20], often not suited for studies at finer resolutions (as in our case). A potential solution to overcome this resolution problem can be obtained by using the link between rainfall and NDVI, as illustrated in the former paragraph, to reconstruct rainfall patterns. Previous research showed that the reconstruction of (historical) precipitation patterns based on established relationships between precipitation and NDVI can give satisfactory results [17,21]. More specifically, the seasonal and interannual variability of rainfall, can be generally well reconstructed and appears clearly from the analysis.

Bubonic plague is caused by the bacteria *Yersinia pestis*. Fleas serve as a vector for spreading the disease, and rodents are the natural hosts [22,23]. Humans are only rarely infected, but due to their large susceptibility to the disease, once infected, mortality is much higher than in rodent populations. In many plague-infected areas, the disease seems to exhibit a seasonal pattern. A few studies in Vietnam and the USA have established statistically that climate has a strong influence on the epizootics, the stage of the plague cycle in which the disease is the most infectious [24-27]. Most of these studies found a positive correlation between human plague cases and rainfall. Cavanaugh [24] investigated the main properties through which precipitation and temperature have an influence. First, these factors have a regulating effect on the population density of fleas, and second, on the efficiency of disease transmission of the fleas. These two factors, population density and vector efficiency, are important regulators leading to human plague epidemics.

In this paper, we aim to (1) establish a relationship between vegetation indices and actual precipitation values; (2) use this relationship to reconstruct spatial and temporal precipitation patterns in northeastern Tanzania at a finer resolution (1 × 1 km, or ~0.01 × 0.01°) than what is provided by TRMM (0.25 × 0.25° or ~25 × 25 km); and (3) in the scope of plague research, investigate the influence of rainfall patterns on plague occurrence. More specifically, we want to establish a relationship between precipitation and greenness characteristics through the follow-up of multitemporal sequences of vegetation indices (MODIS NDVI). The ultimate goal is apply the results of this modelling study on rainfall variability and seasonality at a 1 × 1 km scale in a research in which we aim to find ecological explanations for the distribution of human plague in northeastern Tanzania [28,29].

## Results

### Correlation between precipitation and NDVI

**Figure 1:**
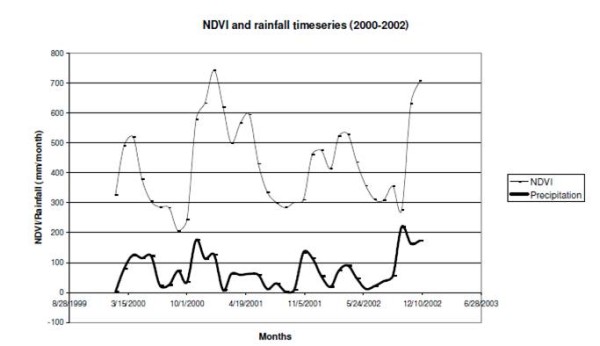
**Comparison of NDVI and precipitation time series of one selected location (4.4°S 38.4°E)**. NDVI values are unitless and indicated with a thin line. The thick line represents the precipitation values in mm/month.

To test the correlation between precipitation and NDVI, we compare graphically the temporal sequences of NDVI and precipitation as an explorative test of a relationship (Figure 1). Seasonal peaks of rainfall are followed by the seasonal peaks of NDVI. Higher rainfall rates, as in March 2000 or September 2002, results in higher NDVI peaks, usually after a time lag of one or two months, which can be explained by the delay in the vegetation water uptake.

Correlations between TRMM precipitation and NDVI were tested at different time lags. A time lag of one month appears to lead to the highest correlations in most of the scenarios except for the *agriculture *scenario (Table 1). Correlations between precipitation and vegetation indices from the same month, or with a time lag of two or three months are lower. This suggest that it takes approximately one month before the effects of rainfall start to appear in the vegetation, probably due to the delay of soil moisture recharge and vegetation growth.

**Table 1:** Spearman correlation coefficients for NDVI-precipitation correlation for different time lags (columns) and different land cover/elevation scenarios (rows)

	Concurrent months	One month	Two months	Three months
**Control**	0.55*	0.80*	0.65*	0.36*
**Agriculture**	0.52*	0.51*	0.79*	0.64*
**Forest**	0.17	0.43*	0.38*	0.24
**Savannah**	0.64*	0.83*	0.68*	0.39*
**<1000 meters**	0.65*	0.82*	0.62*	0.32*
**>1000 meters**	0.45*	0.76*	0.66*	0.37*

Note: The significance of these correlation coefficients have been tested under H_0 _: R^2 ^= 0. The values that are significant at a 0.99 level, are marked with an asterisk. The values marked in bold are those of the scenarios that are selected for the reconstruction of precipitation patterns.

Comparing the highest correlation coefficients of each land cover/elevation scenario with the control scenario (R_2 _= 0.80), it appears that a stratification into land cover or elevation classes does not result into significantly stronger correlations. For *agriculture *(R_2 _= 0.79) and *forest *(R_2 _= 0.43), and *altitudes above 1000 meters *(R_2 _= 0.76), Spearman correlation coefficients are even below the correlation coefficient of the control scenario (R_2 _= 0.80). Contrarily, looking at the NDVI-precipitation relationship for *savannah *(R_2 _= 0.83), and for *altitudes below 1000 meters *(R_2 _= 0.82), correlations between precipitation and NDVI seem stronger than in the control scenario.

### Modelling precipitation from NDVI

The three scenarios with the highest correlation coefficients (control, *savannah *and <*1000 meters*), all at a one month time lag, were selected for further analysis (Table 1). A linear regression line was fitted at this time lag, which resulted in linear equations for estimating rainfall based on an NDVI image between the two variables (Table 2). Correlations are strong, and thus, linear regressions are satisfactory to reconstruct rainfall patterns for the study area, also because rainfall variability is large here.

**Table 2:** Regression equations of the selected scenarios

Scenarios	Linear regression equation	**R**^**2**^
Control scenario	*P*_*estimated *_= 0.434*NDVI - 202.1	0.80
Savannah	*P*_*estimated *_= 0.277*NDVI - 87.37	0.83
<1000 meters	*P*_*estimated *_= 0.326*NDVI - 95.7	0.82

To actually estimate rainfall patterns, we chose the NDVI-precipitation relationships which show the strongest correlations in Table 1 i.e. the scenarios with a one month time lag. In other words, precipitation estimations (mm/month) of month *t *are based on NDVI values of month *t+1*. Since we have MODIS NDVI data from February 2000 until January 2009, we reconstructed monthly rainfall rates between January 2000 and December 2008.

The three scenarios with highest correlation coefficients (control, *below 1000 m *and *savannah*) were tested for their performance on estimating rainfall patterns. Finally, model validations were performed by comparing the predictions with in-situ measured rainfall data obtained from three meteorological stations (Same, Tanga and Lyamungu). In all three cases, the scenario that discriminates for altitude performs the best, as proven by the correlation coefficients (Lyamungu: R^2 ^= 0.26, Same: R^2 ^= 0.66, Tanga: R^2 ^= 0.57; Table 3).

**Table 3:** Spearman correlation coefficients of the comparison of modelled rainfall (mm/month) with measured rainfall (mm/month) in Lyamungu, Same and Tanga

	Tanga	Lyamungu	Same
Control	0.44*	0.09	0.42
Below 1000 m	0.57*	0.26	0.66*
Savannah	0.52*	0.28	0.64*

Note: The significance of these correlation coefficients have been tested under H_0_: R^2 ^= 0. The values that are significant at a 0.99 level, are marked with an asterisk.

### Validation of the model

**Figure 2:**
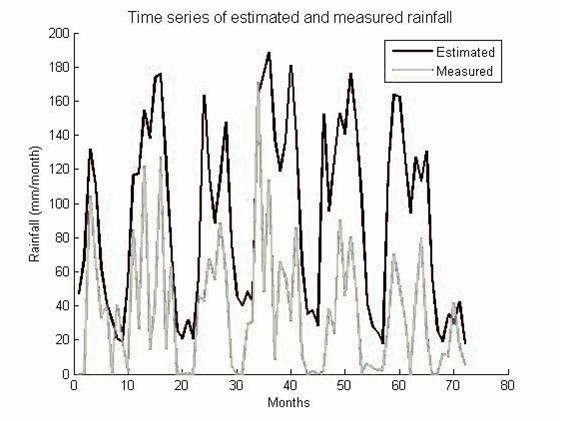
**Comparison of estimated and measured rainfall over a period of 8 years (2000-2008)**. The estimated rainfall is indicated in black, the measured rainfall with a grey line.

A comparison between measured rainfall time series and modelled rainfall time series is presented in Figure 2. The magnitude of rainfall is not predicted accurately: the rainfall values are underestimated. However, interannual and interseasonal trends are visible. The rainy seasons fall at the same time in the estimated and measured rainfall time series, as can be seen in the coinciding peaks. Also, measured high rainfall rates are shown in our estimated rainfall. For example, the higher rainfall rates (180 mm/month) in the beginning of 2003 (starting from month 36) result in high peaks of estimated rainfall (160 mm/month).

### Relation between plague and rainfall

**Figure 3:**
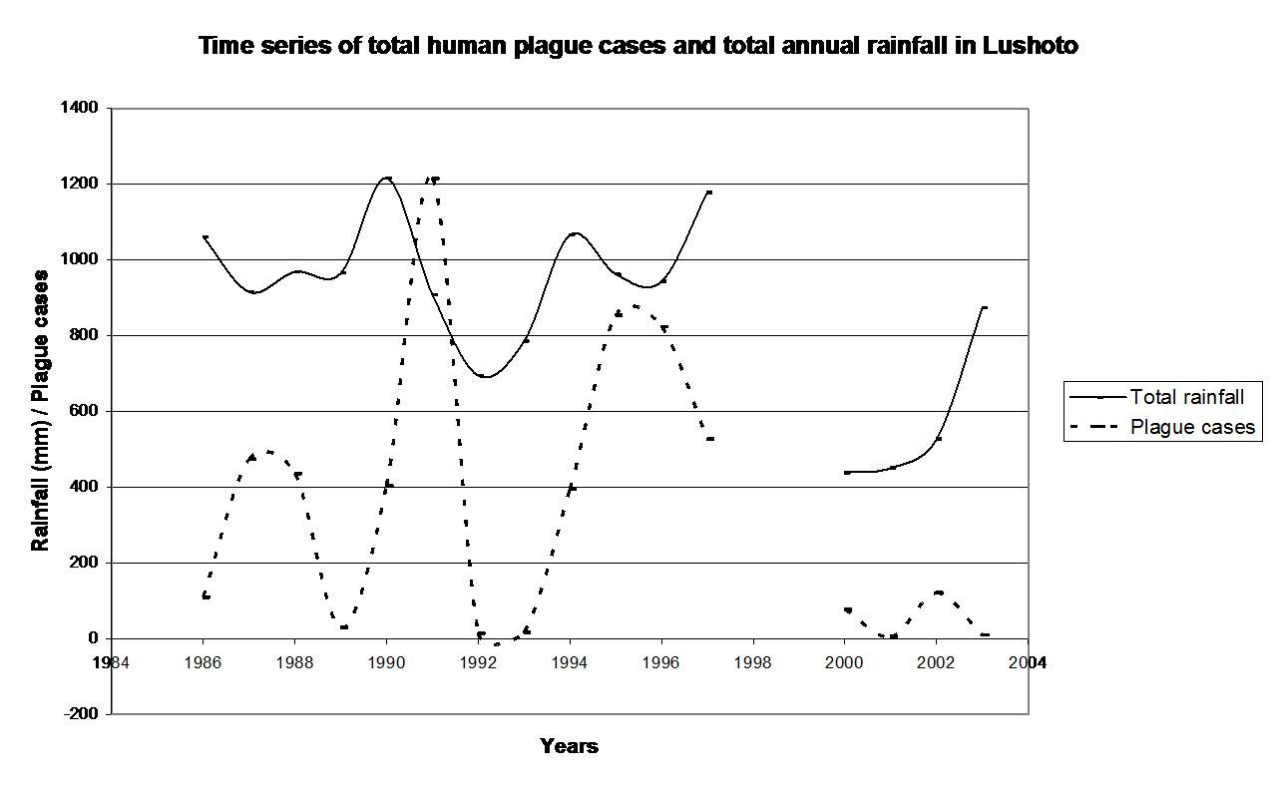
**Annual rainfall and annual human plague cases in the Lushoto district over the period 1986-1997 and 2000-2003**. Annual rainfall is indicated with a full line and is in mm/year units. Annual plague cases are shown with a striped line.

Finally, these time series are extended to earlier years (1986 and 1987) to obtain a more complete image of the influence rainfall has on the occurrence of bubonic plague in our study area. In Figure 3 the course of total plague cases over the years is compared with the total rainfall over those years for the period 1986-1997 and 2000-2003.

From this figure, it appears that rainfall has a positive influence on the occurrence of human plague. Visually, wet years, such as 1986, 1990 and 1994 are followed by peaks in the plague cases one year later. Furthermore, low plague incidences are preceded by dry years, like 1991 and 1992. The correlation coefficients confirm this positive relationship: a correlation coefficient of 0.48 (p = 0.06) is obtained. However, taking a one year time lag into account results in a weaker and insignificant correlation of 0.2 (p = 0.49).

**Figure 4:**
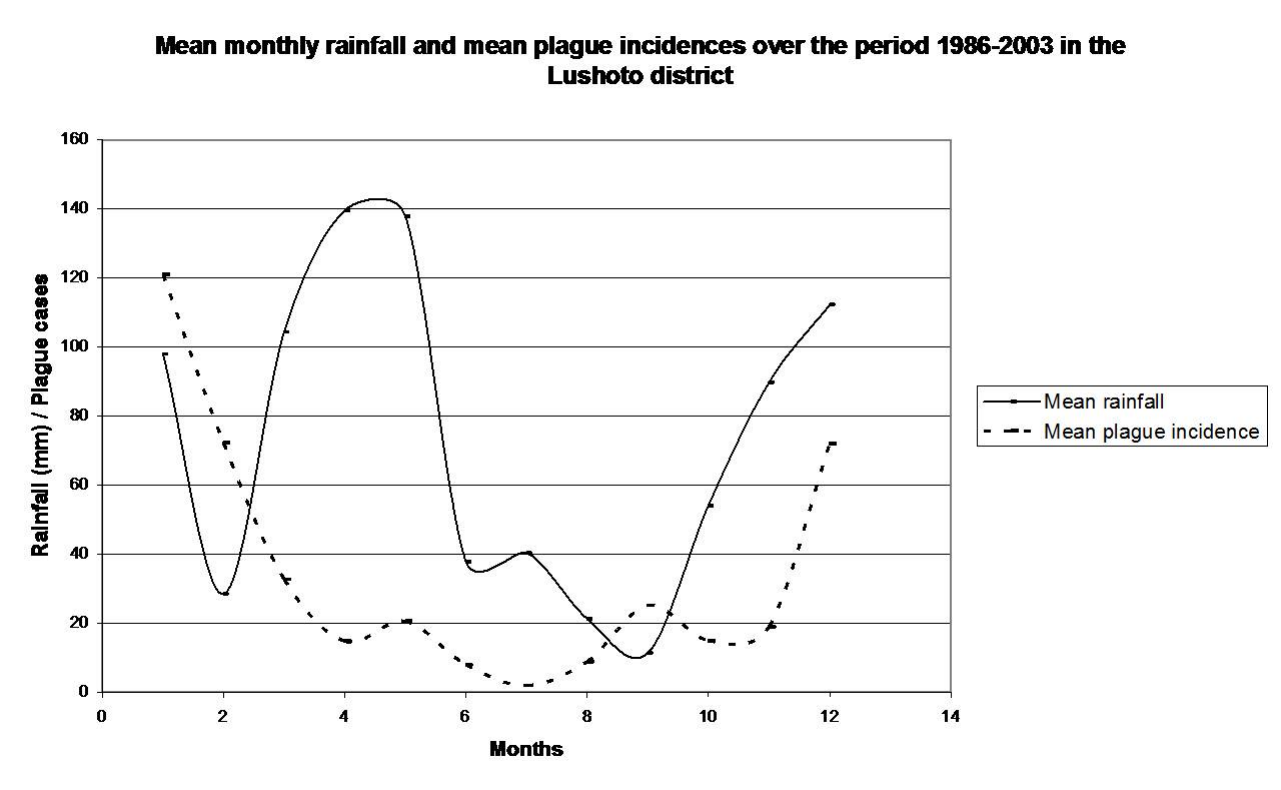
**Mean monthly rainfall and mean monthly human plague cases in the Lushoto district**. Mean monthly rainfall is shown as a full line and values are in mm. These mean values consist of modelled rainfall rates from 2000 to 2003, and measured rainfall rates from 1986 to 1997. The mean monthly human plague cases, taken over the years 1986-2003, are shown as a striped line.

The analysis at monthly scale is conducted with mean values, both for rainfall and number of plague cases, to get a better insight in the seasonality of the occurrence of the disease and to even out the large variations. The results are displayed in Figure 4.

The course of human plague incidences over the year is clear: most cases occur in December, January and February. This coincides with the period just after the short rains of October-December. Again, precipitation seems to be positively correlated with plague occurrence (Table 4). The exception to this positive relationship is the long rainy season from March to May: during and after it, no rise in human plague cases is found.

**Table 4:** Spearman correlation coefficients of the relation between monthly total rainfall and monthly total plague cases in the Lushoto district

Concurrent months	One month time lag	Two months time lag	Three months time lag
0.19	0.33	0.82*	0.57

Note: Values that are significant at a 0.95 are indicated with a *.

## Discussion

The resulting correlation coefficients between precipitation and NDVI are higher in this study than most of those reported in other research on the same subject. There are several possible explanations for this: on the one hand, we use MODIS coverages as vegetation indices and TRMM as precipitation proxies to build the models on, and on the other hand, we test correlations using Spearman correlation coefficients instead of Pearson correlation coefficients. The majority of former but similar research papers on the relationship between vegetation indices and precipitation were conducted using NOAA AVHRR NDVI imagery instead of MODIS [12,19,30]. Many researchers have found that the MODIS sensors show better performance compared to the AVHRR sensor on the issues of atmospheric correction, spectral bandwidth and compositing algorithm [2]. The lack of these improved algorithms in AVHRR leads to reduced correlation coefficients [31]. Reasons why correlation coefficients are higher when using TRMM data instead of in situ measurements; however, higher correlation coefficients were also found by Hermann *et al*. [31]. Indeed, TRMM reflects the average rainfall in the overall area better, since it does not rely on point measurements, but provides accurate rainfall rates in a regularly spaced grid at a higher resolution than what is usually available through in situ measurements [20]. We can therefore execute the analysis in every location of the study area, allowing us to choose representative locations for the sampling of NDVI values, instead of being dependent on the locations of the meteorological stations.

Correlation coefficients for Lyamungu are consistently lower than these for Same and Tanga. Since Lyamungu lies on the southwestern slope of Mount Kilimanjaro, it receives much more orographic rainfall than the other two stations, who get more convective precipitation. The large amounts of orographic rainfall in Lyamungu are not reflected in the NDVI patterns, since vegetation only takes up rain up to a certain amount. Also, much of the heavy rains result in surface run-off, and that rainfall is therefore also not taken up by vegetation. Therefore, we only recommend this technique for convective rainfall areas in the tropics, since in these areas, vegetation is highly dependent on the amount of rainfall. Studies [32,33] show that water as a limiting factor has the most influence in areas with low organic content in the soil, such as in the Kalahari, which consists for the most part out of arenosols [17]. Grist *et al*. [17] sets the limit for a proper reconstruction of precipitation patterns at an annual rainfall of 650 mm, so this method would not work in areas with a higher annual precipitation. However, Amissah-Arthur [34] finds that environmental variation in Kenya was mainly influenced by rainfall, so the reverse process may also yield some good results in areas with more rainfall. Davenport and Nicholson [35], who investigated this relationship in East Africa, find the limit to be a little higher, at 1000 mm annually. Too little rainfall also adversely affects the outcome, due to prematurely wilting of the vegetation cover during droughts [17]. Most likely, further improvements can be made in more water-limited areas, where vegetation is even more linked to the amount of precipitation.

However, the models do not capture magnitude information at the same precision level. Grist and co-authors [17] formulate a number of reasons why errors might occur. First, the relation between vegetation and precipitation is indirect, soil moisture would be a better indicator. This error primarily affects the response of NDVI on high precipitation values, because water ceases to be the limiting factor from a certain rainfall amount on. Second, the assumption of homogeneity when creating the relationship between NDVI and precipitation is biased, and can thus be considered false. Third, the geometric and radiometric errors in the data used, both in ground data and satellite data, can lead to interpretation errors. To minimize these errors in our study, we use satellite data at a resolution of 1 km (MODIS NDVI), and TRMM data instead of point measurements of precipitation. Also, land cover and elevation are integrated in the analysis to obtain NDVI-rainfall relationships in homogeneous areas.

Further improvements can be made by computing the model with other statistical techniques, such as geostatistics. The correlation coefficients in this study were calculated using a normal linear regression technique. However, precipitation and vegetation are variables with a strong spatial component. Knowing rainfall and vegetation values of neighbouring pixels, it is possible to estimate that value for the pixel under investigation. Some authors [8,16,36] have proposed geostatistics to get better estimates of the magnitude of the precipitation-vegetation relationship. The assumption for this better estimate is twofold: precipitation data, and in a lesser extent NDVI, show deviations from the normal distribution, and have a mean and variance that change gradually, indicating non-stationarity of the data. These two properties, normality and stationarity, are assumed to be correct when using the linear regression technique. Since this is not the case here, linear regression may lead to overestimations of the correlation coefficient due to inaccurate standard deviations [37,38]. Eklundh [16] states that ordinary least squares regression overestimated the correlation coefficient with 50%. Foody [36] used a regression with geographical weights in north Africa and the Middle East, and found a better correlation coefficient (0.96) than using a linear regression (0.67) for the same data. Finally Hermann *et al*. [31] state that using ordinary linear regression does not affect the spatial pattern of these correlations although spatial autocorrelation does have an influence on the magnitude of the correlation coefficients.

This rainfall modelling study was carried out in the framework of a research in the Western Usamabara Mountains in which we investigate the influence of rainfall variability on human plague occurrences. Results show that the annual plague cycle, which is characterized by a yearly peak in the number of cases in January, is indeed related to the annual cycle of the rainy seasons. At a two month time lag between the onset of the short rainy season and high number of plague cases, a correlation coefficient of 0.82 was found [39]. This confirms the theory of a positive relation between rainfall and plague cases like shown in other research [24-27]. Interannual trends also seem to play a role, with wet years (1986, 1990 and 1994) followed by peaks in the number of plague cases one year later. Furthermore, low plague incidences are preceded by drier years (1991 and 1992). However, this positive relation does not indicate a causal relation between the two, since the cycle of the disease is complex: changes in the environment can affect the flea as vector of the disease, some specific mammals as their host and the transmission efficiency of the disease. Since they all interact differently with their surroundings, wetter and dryer years affect them in other ways. For example, the number of fleas increases due to the increased soil moisture up to a certain threshold, after which flea reproduction is suppressed by the high soil moisture levels [27]. Also, precipitation patterns influence many other environmental characteristics, such as plant reproduction, which in turn have their impact on the distribution of bubonic plague.

## Conclusions

In this paper, we aimed to expand time series of rainfall data in time and space at a fine resolution (1 km), to allow for a better analysis of phenomena influenced by precipitation patterns when locally recorded rainfall data are not available. The model for rainfall estimation is based on the relationship between vegetation, as presented by MODIS monthly NDVI composites, and rainfall, as measured by the TRMM, and was tested using locally recorded rainfall time series from three locations in the study area. We conclude that the relationship between rainfall (TRMM) and NDVI (MODIS) is strongest after a one month time lag in northeastern Tanzania and that this relationship can be used to reconstruct rainfall patterns at 1 × 1 km resolutions. Improvements are made by repeating the analysis with different land cover classes and elevation classes. These results are found to be useful for extending a series of rainfall data over time and in space, to investigate the influence of rainfall on plague occurrence. Results show that occurrence of bubonic plague is positively correlated with rainfall at a two months lag.

## Methods

### Study area

**Figure 5:**
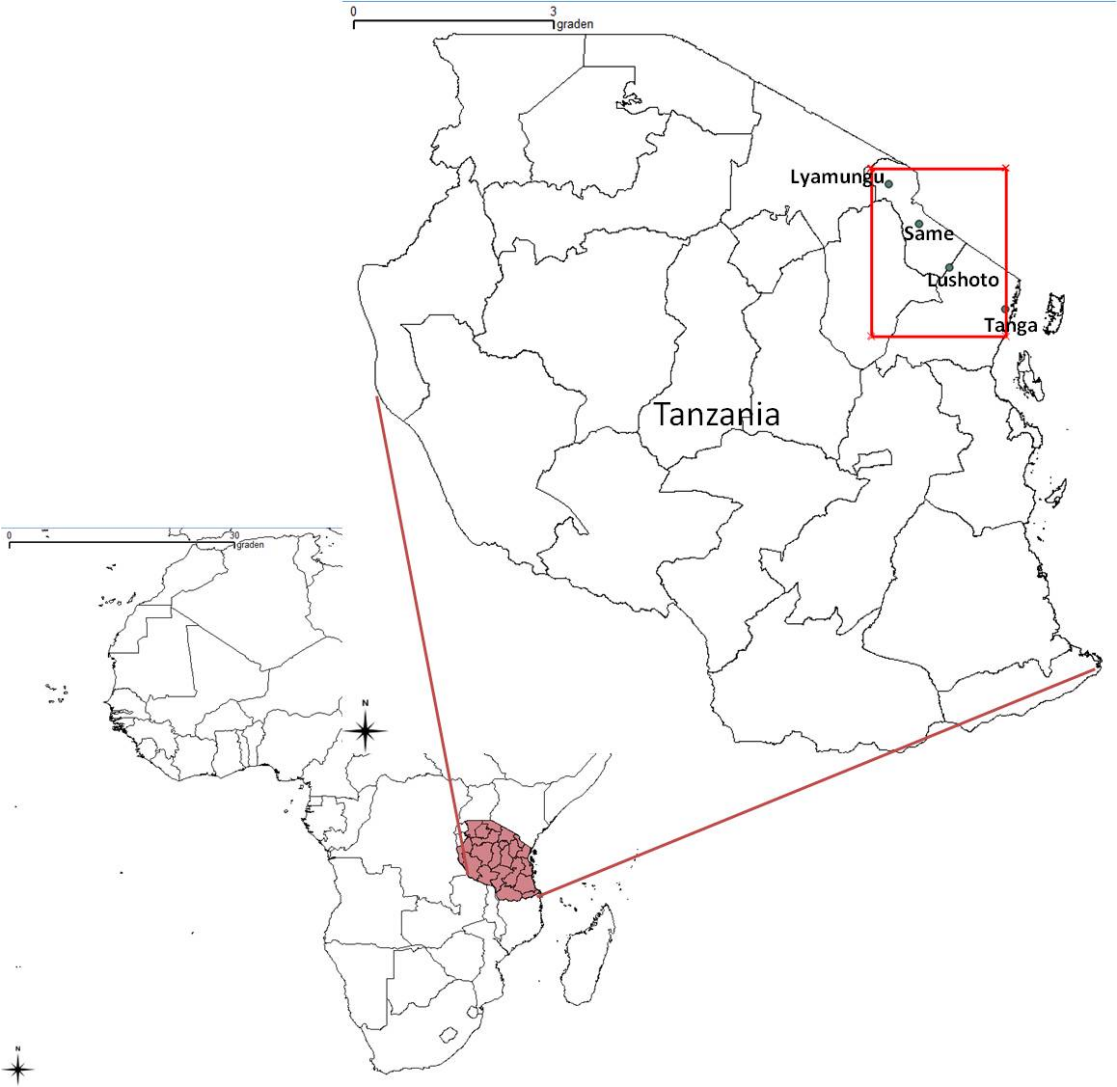
**Map locating Tanzania and the study area of this research**. Districts of Tanzania are shown with black lines. The study area is located within red rectangle and four meteorological stations from which data were used in model validation are annotated with black dots.

Our study area lies on the eastern coast of Central Africa and encompasses the Usambara and Pare Mountains, and the Maasai plains, situated in the northeast of the United Republic of Tanzania (Figure 5). The boundaries of the study area are set at 3-5.5°S and 37-39°E. The three meteorological stations from which rainfall time series were used in the model validation, Same (3.83°S 37.72°E, 1100 m), Lyamungu (3.23°S 37.25°E, 1250 m) and Tanga (5.1°S 39°E, 60 m), are indicated as well. The Usambara and the Pare Mountains are both historical plague regions. The last human outbreak in the Pare Mountains occurred in 1964 (Kilonzo, Pers. Comm.), while >6000 human plague cases were reported from villages in the Usambara Mountains between 1980 and 2004 [28]. It was found that plague incidence differs from year to year, and among villages. Even though control measures have been taken, human plague returned every year. This led to the hypothesis that there are some ecological factors responsible for plague occurrence [40].

The study area consists of mountains (i.e. the Usambara and Pare Mountains, elevation range between 750 and 2630 m [41]) and plains (i.e. the eastern part of the Maasai plains), and differences in land cover can be distinguished. On the Maasai plains, extensive cultivation is possible because of the potential large size of cultivated fields. Agriculture is alternated with savannah and pastoral rangelands, although the latter are disappearing to be transformed to cultivated land [42].

In the Pare and Usambara Mountains, a greater variety of land cover types exist, which is evidently influenced by the topography, i.e. the position in the landscape. Moreover, land cover types are also affected by climate, which in turn is influenced by the proximity of the Indian Ocean and the prevailing easterly winds [43].

In northeast Tanzania, two rainy seasons exist: the short rains from October to December, called *mvuli*, and the long rains from March to May, called *masika *[44]. These rain seasons are associated with the seasonal movement of the Intertropical Convergence Zone (ITCZ), which moves northwards during the long rains, and southward in the October-December period [44]. The total rainfall in northeast Tanzania is highly affected by the topography. In lowland areas and on the rainshadow sides of the mountains, yearly precipitation amounts to 600 mm, whereas in the highlands, an average of up to 1200 mm can fall [45]. Due to the interaction of major factors, i.e. large altitude differences, various vegetation patterns and the proximity of the Indian Ocean, a well pronounced spatial and temporal precipitation pattern exists in northeastern Tanzania [43].

### Data

To develop a model for estimating rainfall patterns based on NDVI data, we use monthly NDVI composites of MODIS and TRMM *Monthly Gridded 0.25° × 0.25° merged TRMM and other sources estimates (3B43) *of the period February 2000 until January 2009. Monthly composited NDVI time series of MODIS at a spatial resolution of 1 km [46] are used to estimate rainfall patterns, at a higher resolution than that the existing rainfall rates of TRMM (0.25°).

Monthly rainfall rates of meteorological stations in Same, Lyamungu and Tanga and recorded by the Tanzania Meteorological Agency, are used to validate the model. Rainfall rates for these stations are available for the period 1980-2005.

Because of the heterogeneity of the study area that can influence relationships between NDVI and precipitation, stratification based on land cover and altitude is implemented. Since our study area is heterogeneous in these aspects, they can influence the NDVI-precipitation relationship. We want to see if differentiation in the regression analysis is needed, and if the model can be improved by looking at different land cover types or at different altitudes. Therefore, we create a land cover map, based on Landsat ETM+ imagery, and a DEM based on SRTM [47]. Seven images are mosaicked to obtain a full map of the study area, for SRTM and Landsat, and the Landsat images are atmospherically corrected using the cosine approximation (COST) algorithm in Idrisi Andes. Land cover mapping is based on maximum likelihood classification. Three land cover classes are distinguished, (1) agriculture, (2) savannah and (3) forest. Other classes, i.e. water, clouds, shadow and bare soil are also taken into account in the land cover classification, so that these regions can be masked and thus left out of the analysis. Much research has been performed on the persistent plague focus in the Lushoto district in the western Usambara mountains. Long-term data (1986-2003) on plague occurrence are available for that district in the form of individual hospital records. These data are used to examine the influence of precipitation on the occurrence of the plague.

In order to investigate the plague-rainfall relationship between 1986 and 2003, additional mean monthly rainfall data of Lushoto have been made available by SECAP, for the period 1986-1997. Moreover, simulated rainfall data are obtained from Landsat data of October and December 1986, and January 1987. These are radiometrically calibrated, atmospherically corrected, converted to NDVI values and resampled to match the 1 km grid of MODIS, and then used in our model to estimate rainfall rates.

### Model calibration

We developed a model based on a linear regression between monthly rainfall from TRMM and monthly NDVI data. Five regression scenarios (based on either land cover classes or elevation classes, Table 5) were defined based on the stratification into land cover and altitude classes.

**Table 5:** Regression scenarios based on land cover and elevation

Stratification into land cover classes	Agriculture Savannah Forest
**Stratification into elevation classes**	<1000 meters
	>1000 meters

To check the validity of this stratification, a control scenario that does not discriminate between land cover or altitude, was included in the analysis. Based on this stratification, NDVI and rainfall pixels of every month were sampled within the appropriate land cover classes. These sampled values were averaged per month. For each scenario, we consequently had 96 data points of NDVI and rainfall values, with which linear regression analysis was executed.

For the actual analysis, four timing scenarios were defined, since a time lag can significantly influence the correlation between a vegetation index and precipitation [11,12,16]. These time lags vary from one to three months. At each time lag, precipitation was tested for its correlation to monthly NDVI. The linear regression line was obtained by comparing NDVI with rainfall at an optimal time lag, as indicated by the Spearman correlation coefficient.

### Model validation

The linear regression model obtained during the calibration was used as the model equation for calculating precipitation:

$$P_{estimated}=a+b*NDVI$$

To validate the model, estimated monthly rainfall values calculated from MODIS NDVI images were compared with measured rainfall rates of meteorological stations for the same months. In every monthly raster image of estimated rainfall, the rainfall rates at the locations of every meteorological station were extracted. These monthly estimated rainfall rates were then compared with the monthly in situ measurements of rainfall. The validation measures were again Spearman correlation coefficients.

### Plague-rainfall relationship

For the Lushoto district, measured monthly rainfall data are available for the period 1986-1997. These data are complemented by the modelled rainfall rates from 2000 to 2003 based on MODIS NDVI data, and from October and December 1986, and January 1987, estimated from Landsat TM data. The modelled rainfall rates of October and December 1986 and January 1987 are based on atmospherically corrected, calibrated Landsat TM images of the Lushoto district which are converted to NDVI and resampled to a 1 km grid. These Landsat data were selected for high radiometric quality and absence of clouds. The monthly NDVI composites of MODIS are based on maximal NDVI values rather than mean values, so we assume that these Landsat data are representative for the month in which they have been taken.

Analyses at two temporal scales are made to get a better insight of the influence of precipitation on plague occurrence: one based on annual rainfall and annual plague cases for 1986-1997 and 2000-2003, and one with monthly rainfall and monthly number of human plague cases. Spearman correlation coefficients are calculated to check the strength of the precipitation-plague relationship. If necessary, a time lag is incorporated, since we expect to see some delay in the response of the environment to precipitation, and of plague outbreaks to changes in the environment.

## Competing interests

The authors declare that they have no competing interests.

## Authors' contributions

All authors have read and approved the final manuscript. AD conceived and designed the study, and drafted the manuscript. SN helped to draft the manuscript and had supervision over the entire study. HG and DK defined and facilitated the research subject, have followed-up closely its progress, and contributed to the manuscript revision.
